# Extra-gastrointestinal stromal tumor of the pancreas: case report and review of the literature

**DOI:** 10.1186/1477-7819-12-105

**Published:** 2014-04-23

**Authors:** Valentina Beltrame, Mario Gruppo, Davide Pastorelli, Sara Pizzi, Stefano Merigliano, Cosimo Sperti

**Affiliations:** 1 Department of Surgery, Oncology and Gastroenterology, 3rd Surgical Clinic, University of Padua, Giustiniani 2, 35128, Padua, Italy; 2 Department of Oncology, Rare Tumors Unit, Veneto Institute of Oncology, Padua, Italy; 3 Department of Pathology, University of Padua, Padua, Italy

**Keywords:** Differential diagnosis, Gastrointestinal stromal tumor, Pancreas, Pancreatectomy, Pancreatic neoplasms

## Abstract

Primary extra-gastrointestinal stromal tumor (EGISTs) arising in the pancreas is extremely rare: only 20 cases have previously been reported in the English literature from 2000 to 2013. We reported a case of EGIST of the pancreas in a 69-year-old woman who presented with abdominal pain and with a solid, heterogeneously enhancing neoplasm in the uncinate process of the pancreas, revealed preoperatively by an abdominal computed tomography scan. A diagnosis of neuroendocrine tumor was suggested. Positron emission tomography with 68Ga-DOTATOC did not show pathological accumulation of the tracer in the pancreas. The patient underwent enucleation, under ultrasonic guidance, of the pancreatic tumor that emerged to the surface of the pancreas. Histopathology and immunohistochemical examination confirmed the final diagnosis of EGIST of the pancreas (CD117+), with one mitosis per 50 high-power fields. Although rarely, GIST can involve the pancreas as a primary site, and this tumor should be considered in the differential diagnosis of pancreatic neoplasms.

## Background

Gastrointestinal stromal tumors (GIST) are the most common mesenchymal tumors of the gastrointestinal tract, with an annual incidence of 10 to 20 per million [1]. GISTs are neoplasms arising from, or differentiating along, a line similar to the gastrointestinal pacemaker cells, the interstitial cells of Cajal (ICCs) [2-4]. ICCs form a network around the myenteric plexus and within the muscolaris propriae of the gastrointestinal wall. GISTs may occur in the entire length of gastrointestinal tract from the esophagus to the anus; however, the most common sites are stomach (60%), small intestine (30%), rectum (5%), and esophagus (<5%) [1]. Duodenal GISTs constitute 30% of primary duodenal tumors and less than 5% of gastrointestinal stromal tumors [5-7]. Sometimes, these tumors may arise from the omentum, mesentery, gallbladder, and retroperitoneum, adjacent, but separate from the stomach and the intestine [8-10]; in this case the neoplasm is defined as ‘extra-gastrointestinal stromal tumors’ (EGISTs). EGISTs do not display connection to the wall or the serosal surface of the viscera. EGISTs arising in the pancreas are extremely rare, and only 20 cases have been reported in the literature from 2000 to 2013 [11-31]. We present a new case of a pancreatic EGIST misinterpreted as non-functioning endocrine tumor, in a 69-year-old woman. A review of the literature is also included.

## Case presentation

**Figure 1:**
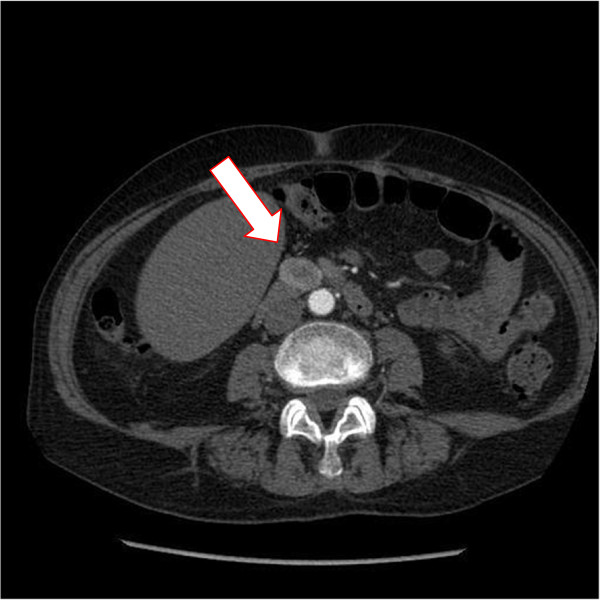
Abdominal CT scan showing a 2-cm, contrast-enhanced mass (arrow) in the uncinate process of the pancreas.

**Figure 2:**
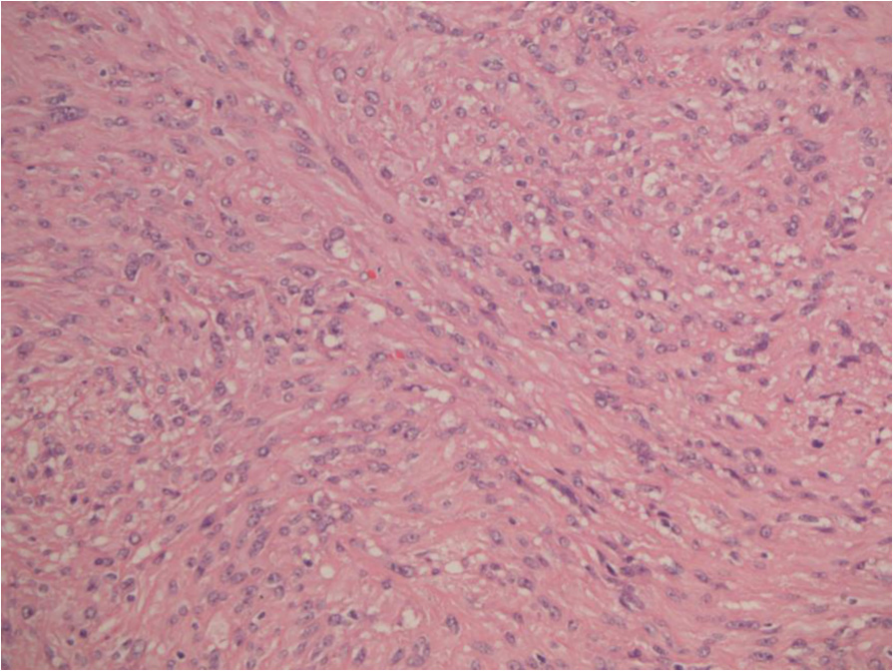
Stromal tumor composed of spindle and epithelioid cells with focal vacuolar (signet ring) change. (E & E, 20×).

**Figure 3:**
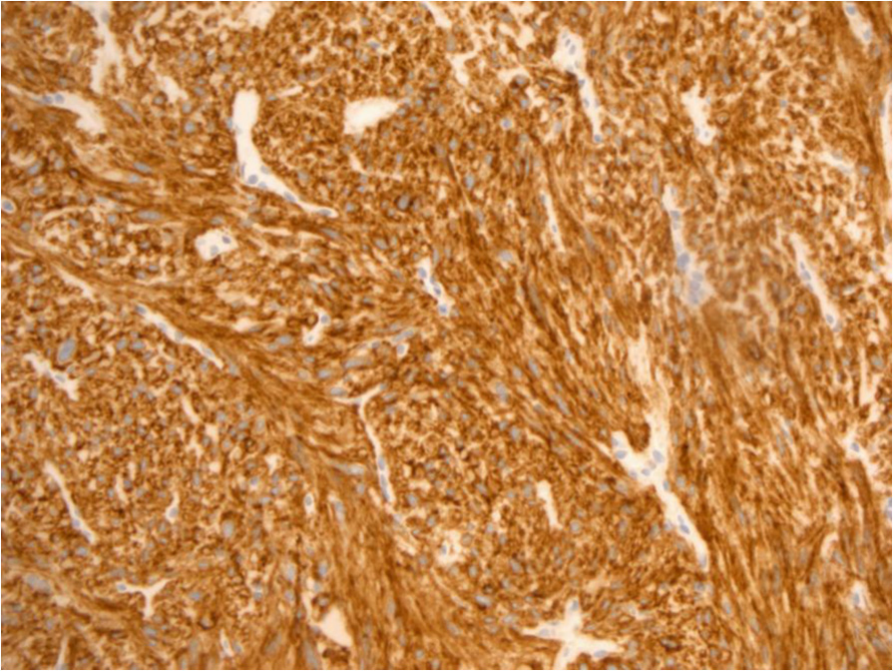
Immunostain for c-KIT: strong and diffuse cytoplasmic immunoreactivity.

**Figure 4:**
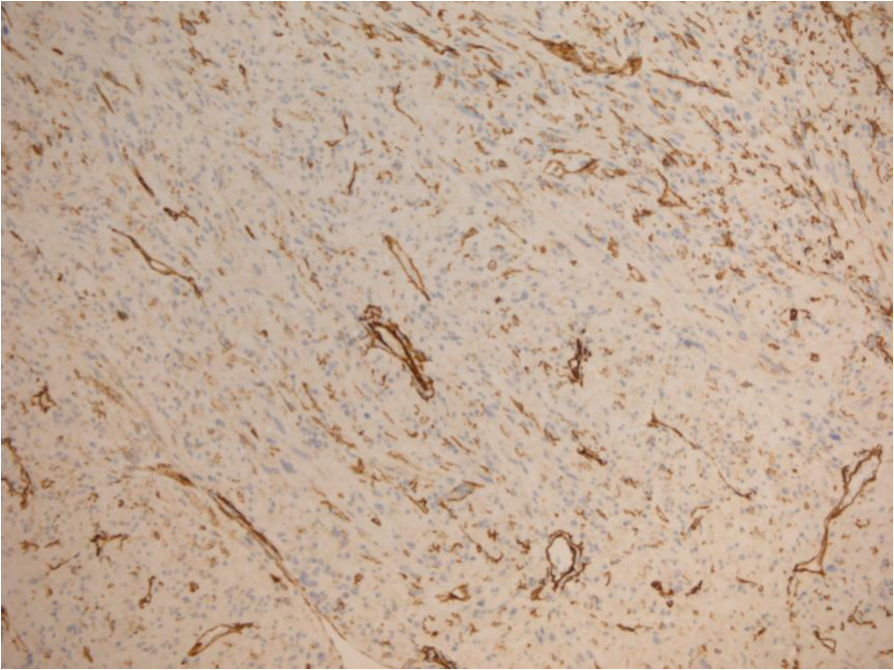
Immunostain for CD34: focal immunoreactivity.

A 69-year-old woman presented in March 2013 with abdominal pain localized in the right hyphocondrium. There was no history of vomiting, gastrointestinal bleeding, jaundice, anorexia, or weight loss. Abdominal ultrasonography did not show pathologic features, but the pancreas was not clearly visualized. Contrast-enhanced computed tomography (CT) of the abdomen (Figure 1) revealed a solid, hypervascular nodule in the uncinate process of the pancreas, measuring 22 × 15 mm. The possibility of a neuroendocrine tumor was considered; therefore she underwent Gallium-68 somatostatin receptor positron emission tomography (PET) (68Ga-DOTATOC), without evidence of neoplasms with pathologic expression of somatostatin receptors. Routine laboratory investigations, exocrine and endocrine serum markers, and hormonal panel were within normal limits, except for CEA: 5.7 ug/L ( reference value <5 ug/L). Endoscopic ultrasound (EUS) confirmed a 2-cm hypoechoic tumor in the head of the pancreas; fine-needle aspiration of the lesion was not available at that moment. At laparotomy, in April 2013, a well-demarcated, red nodule was identified in the uncinate process of the pancreas; no attach with the duodenal wall was found. Intraoperative sonography showed that the 2-cm hypoechoic mass was separated from the main pancreatic duct. Careful enucleation of the tumor with Harmonic scalpel, under ultrasound guidance, was successfully performed. Pancreatic capsula was closed with interrupted, absorbable stitches. The postoperative course was uneventful, and the patient was discharged 7 days after surgery. Macroscopic examination showed a 2.4 cm well defined, ovoid mass. Microscopically, the tumor was composed of spindle cells, with focal atypia (Figure 2). The mitotic count was one mitoses/50 high power fields (HPFs). Immunohistochemical examination showed neoplastic cells diffusely positive for antibodies against CD 117 (Figure 3), focally positive for CD34 (Figure 4) and smooth muscle actin, while cells were negative for desmin. A diagnosis of pancreatic GIST with low risk of malignancy has been placed, pT2N0M0, stage I according to TNM (AJCC) classification [32]. On molecular genetic examination, deletion of three nucleotides in exon 11 of c-Kit was found. She did not receive any adjuvant therapy after surgery; 12 months later she is in good general condition and there is no evidence of recurrent disease.

## Discussion

We reported a rare case of a primary pancreatic GIST: the small size, the well circumscribed margins of the lesion, and the contrast enhancement at CT examination suggested a diagnosis of neuroendocrine tumor of the pancreas. GISTs reported outside the gastrointestinal tract as apparent primary tumors are defined as ‘extra-gastrointestinal stromal tumors’ (EGISTs). The concept of GIST has recently been established, due to the progress in immunohistochemical analyses. It is presumed that these tumors originate from the interstitial cells of Cajal (ICCs), pacemaker cells, which are present throughout the wall of the gastrointestinal tract and which regulate the motility. ICCs share many characteristics with EGISTs, including expression of CD117 and CD34 [2]. In fact, the most selective immunohistochemical markers differentiating GISTs from true smooth muscle tumors is the expression of the c-Kit receptor tyrosine kinase (CD117 antigen) in 95% of GISTs. In 2004, Yamamoto et al. [31] reported that EGISTs show similar KIT mutations of typical GISTs suggesting that these tumors have a similar origin. However, at present the origin of EGISTs remains controversial. Some authors believe that GISTs and EGISTs arise from the common precursor cell of ICCs and the smooth muscle cells of the gut, which may account for their growth within and outside the gastrointestinal tract [33]. Other, simpler, explanation suggests that EGISTs are in fact mural GISTs with extensive extramural growth, resulting in eventual loss of their connection with the gut wall [33].

Pancreatic GISTs are extremely rare. We collected only 21 cases (including our patient) from the English literature: their clinicopathologic features and outcomes are summarized in Table 1. The age of the patients ranges from 30 to 84 years, with a mean age of 55.0 years. The male:female ratio was 10:11. Ten of 21 (48%) tumors occurred in the head of the pancreas, five in the tail (34%), four involved both body and tail (19%), and two occurred in the uncinate process (%). EGIST rarely (*n* = 1.5%) involved the entire pancreas [24]. There is a great variation in size (range, 2.4 to 34 cm). The mitotic count was <5/50 HPFs in eight cases (38%) and >10/50 HPFs in four cases (19%). Molecular biology of EGIST has been investigated in only two reports [14,22] and in our patient: two exhibited deletion of base pairs in exon 11, and one showed DNA polymorphism of L862L in exon 18 of c-KIT gene [22]. All but one patient underwent surgery: one patient with metastatic disease died 5 days after admission without operation. One patient underwent palliative operation (cystojejunostomy) and the remaining 17 patients underwent pancreatic resection (in 5 extended to adjacent organs), one had local resection, and one (our case) enucleation of the tumor. Follow-up was available in 19 cases. Disease recurrence following surgery was reported in five cases: three patients underwent re-resection and are alive and free after 30, 41, and 48 months, respectively. Overall, 18 patients are alive with a median survival time of 17.5 months (range, 1 to 66 months). Despite the limited number of cases and the short follow-up time, it appears that resection of pancreatic GISTS may offer a good prognosis, even in recurrent disease.

**Table 1:** Clinicopathologic features, treatment, and outcome of EGISTs reported in the English literature

**Author**	**Year**	**Age (years)**	**Sex**	**Site/Treatment**	**Size (cm)**	**Mitotic count (/50 HPF)**	**Follow-up, months**
Neto et al. [11]	2004	67	F	Body and tail/DP + Imatinib	20	120; CD117(+) CD34(+)	A, Relapse, 1
Yamaura et al. [12]	2004	54	F	Tail/DP	14	Few; CD117(-) CD34(+)	A, NED, 30
Krska et al. [13]	2005	38	F	Body and tail/DP	17	1; CD117(-) CD34(+)	A, NED, 30
Daum et al. [14]	2005	70	F	Head/PD + Imatinib	10	2; CD117(+) CD34(-)	A, NED, 6
Showalter et al. [15]	2008	72	F	Tail/DP	7	3; CD117(+) CD34(-)	A, NED, 27
Yan et al. [16]	2008	47	M	Uncinate process/NR	2.4	3; CD117(+) CD34(-)	NA
Yang et al. [17]	2008	55	M	Body and Tail/DP + Imatinib	NR	NR; CD117(+) CD34(+)	Relapse, 24,- A, NED,41
Harindhanavudhi et al. [18]	2008	63	F	Body/Cystojejunostomy + Imatinib	16	<5; CD117(+) CD34(+)	NA
Trablesi et al. [19]	2009	52	F	Head/PD	10.5	6; CD117(+) CD34(+)	A, NED, 10
Goh et al. [20]	2009	58	M	Head/PD	9	>10; CD117(+); CD34 (-)	A, NED, 60
Padhi et al. [21]	2010	42	F	Body and tail/DP	35	6-8; CD117(+) CD34(+)	A, NED, 10
Saif et al. [22]	2010	31	M	Head/PD + Imatinib	8	48; CD117(+) CD34(-)	A, Relapse, 9
Crisan et al. [23]	2010	61	M	Tail	34	NR	A, NED, 3
Joshi and Rustagi [24]	2010	84	M	Whole pancreas/NOP	34	NR; CD117(+) CD34(+)	Dead day 5 of admission
Rao et al. [25]	2011	40	M	Head and body/PD + Imatinib	6.5	8-10; CD117(+) CD34(+)	Relapse, 24; A, NED 30
Cecka et al. [26]	2011	74	F	Tail/DP	11	<5; CD117(+)CD34(+)	A, NED, 66
Vij et al. [27]	2011	35	M	Head/PD + Imatinib	6.5	12-15; CD117(+) CD34(-)	Relapse, 24; A, NED 48
Soufi et al. [28]	2012	39	M	Head/PD + Imatinib	9	5; CD117(+) CD34(+)	A, NED, 24
Kim et al. [29]	2012	55	M	Tail/DP + Imatinib	15.8	7; CD117(+) CD34(-)	A, NED, 4
Babu et al. [30]	2012	55	F	Head/local resection	5	6-8; CD117(+); CD34 (+)	A, NED, 11
Present case	2013	63	F	Uncinate process/enucleation	2.4	1; CD117(+)	A, NED, 6

A = alive; DP = distal pancreatectomy; NA = not available; NED = not evidence of disease; NOP = not operated; NR = not reported; PD = pancreaticoduodenectomy.

The clinical presentation of EGISTs is variable, depending on the location and size of the tumors. The tumor may even be found incidentally. The most frequent clinical symptoms are: abdominal pain, ileus, bleeding, anemia, and weight loss. Our patient presented with a small, solid, hyperintense lesion resembling a neuroendocrine tumor. A similar finding of small, solid tumor in the uncinate process of the pancreas was reported by Yan et al. [16] in a case of GIST diagnosed by EUS fine-needle aspiration (FNA): unfortunately, biopsy was not available for our patient. Interestingly, 50% (11/21) of the reported cases showed radiologic features of heterogeneously mass (necrotic areas) or solid-cystic appearance; thus, problems in differential diagnosis with cystic neoplasms of the pancreas could be arise. The accuracy of CT determination of the pathological diagnosis of a pancreatic cystic lesion is less than 50% [34,35], but endoscopic ultrasound-guided FNA may be helpful for the diagnosis of pancreatic lesions [36]. The diagnosis of GIST is based on histological, immunohistochemical, and molecular features. Microscopically, this tumor, consisted of spindle cell and epithelioid cells. The cytologic differential diagnosis for these spindle cell proliferations includes leiomyoma, schwannoma, fibromatosis, inflammatory fibroid polyps, and gastrointestinal muscularis sampling [16]. Immunoistochemical positivity of CD117 confirms the diagnosis of GIST. GISTs exhibit a broad spectrum of clinical behaviors, with some low-risk lesions remaining stable for years, while others progress rapidly to metastatic disease. Various parameters are proposed to predict the malignant potential of GIST, such as tumor size, mitotic activity, tumor location, non-radical resection, tumor rupture, peritoneal dissemination, metastasis, and invasion into adjacent organs. National Institute of Health (NIH) consensus criteria (Fletcher’s criteria) [37] proposed risk stratification of tumor behavior into risk categories of very low, low, intermediate, and high risk of metastasis, based on its size and mitotic activity. Tumors larger than 10 cm in size and with more than 10 mitoses per 50 HPF are at high risk of aggressive behavior [33,37]. Standard treatment for primary GIST is complete surgical resection with the aim to obtain negative microscopic margins of resection [13,38,39]. Lymphatic spread of GISTs is uncommon; therefore, a systematic lymph node dissection is not a standard surgical management. In our patient we performed a simple tumor’s enucleation because of a preoperative diagnosis of neuroendocrine tumor. However, after discussion with oncologists, the small size of the lesion and the low-risk according to previously reported prognostic criteria [37], suggested that it was an adequate operation, as reported in a recent study [40]. The patient is alive and free of disease 1 year after operation. The surgical management may offer primary surgery at the time of diagnosis, neoadjuvant chemotherapy followed by surgery, adjuvant therapy after surgery, or debulking surgery in patients with metastatic or advanced disease. Observation only was recommended in case with R0 resection or low-risk GIST. In recent decades, medical therapy for GIST is improved (Imatinib, Sunitinib, Nilotinib, Sorafenib, Dovitinib, and so on) and consequently disease-free survival after surgery is also much improved: in fact, it is recommended in patients with R1 or R2 resection [41]. The results of some clinical trials [42-49] with targeted therapy of GIST are reported in Table 2. Obviously, data about targeted therapy for EGIST are limited. In the review of the literature, among 19 high-risk EGISTs, only nine cases (47%) received adjuvant Imatinib therapy (Table 1).

**Table 2:** Selected agents investigated in the management of GIST

**Author**	**Year**	**Pts (*n*)**	**Phase study**	**Treatment**	**End point**	**Outcome (% or median)**	***P *** **value**
Demetri et al. [42]	2002	147	II	Imatinib	OR	81.6%	
Demetri GD et al. [43]	2006	312	III	Sunitinib/placebo	TTP	27.3 *vs.* 5.4 weeks	<0.0001
Blanke et al. [44]	2008	746	III	Imatinib 400 mg/	PFS	18 *vs.* 20 months	0.13
				Imatinib 800 mg	OS	55 *vs.* 51 months	0.83
De Matteo et al. [45]	2009	713	III	Imatinib/placebo	PFS	98% *vs.* 83%	<0.0001
Dubreuil et al. [46]	2009	30	II	Masitinib	PFS	165.2 weeks	
Sawaki et al. [47]	2011	35	II	Nilotinib	PFS	16 weeks	
Park et al. [48]	2011	31	II	Sorafenib	PFS	4.9 months	
					OS	9.7 months	
Joensuu et al. [49]	2012	400	III	Imatinib 12mo/36mo	RFS & OS	65.6% *vs.* 47.9%	<0.0001
						92.0% *vs.* 81.7%	0.019

OR = overall response; OS = overall survival; PFS = progression-free survival; RFS = recurrence-free survival; TTP = time to tumor progression.

## Conclusion

In conclusion, we report a new case of EGIST arising from the pancreas, misinterpreted as neuroendocrine tumor. Despite its rarity, GIST should be kept in mind in the differential diagnosis of primary tumors of the pancreas, especially when a hypervascular lesion, solid or cystic, is lacking somatostatin receptors at PET examination. Radical resection of pancreatic GISTs is the treatment of choice, and repeat surgery for recurrence may offer a prolonged survival.

## Consent

Written informed consent was obtained from the patient for publication of this case report and any accompanying image. A copy of the written consent is available for review by the Editor-in-Chief of this Journal.

## Competing interests

The authors declare that they have no competing interests.

## Authors’ contributions

VB and CS conceived the study, carried out the literature search, and drafted the manuscript; MG and DP helped in management of the patient; SP carried out the pathologic diagnosis and immunoassays; SM made critical revisions and supervision. All authors read and approved the final manuscript.
